# Supplementary material for: Polymorphisms in Pfkelch13 domains before and after the introduction of artemisinin-based combination therapy in Southwest Nigeria
Source: PLoS One. 2025 Mar 31;20(3):e0316479. doi: 10.1371/journal.pone.0316479 (PMC11957316; doi:10.1371/journal.pone.0316479)
Supplement: Supporting information 2 — (ZIP) [file pone.0316479.s002.zip › 133KN2R_PREMIX_Plate_KELCH2_H01.pdf]

Samples: 11939
Bases: 930
Average spacing: 13

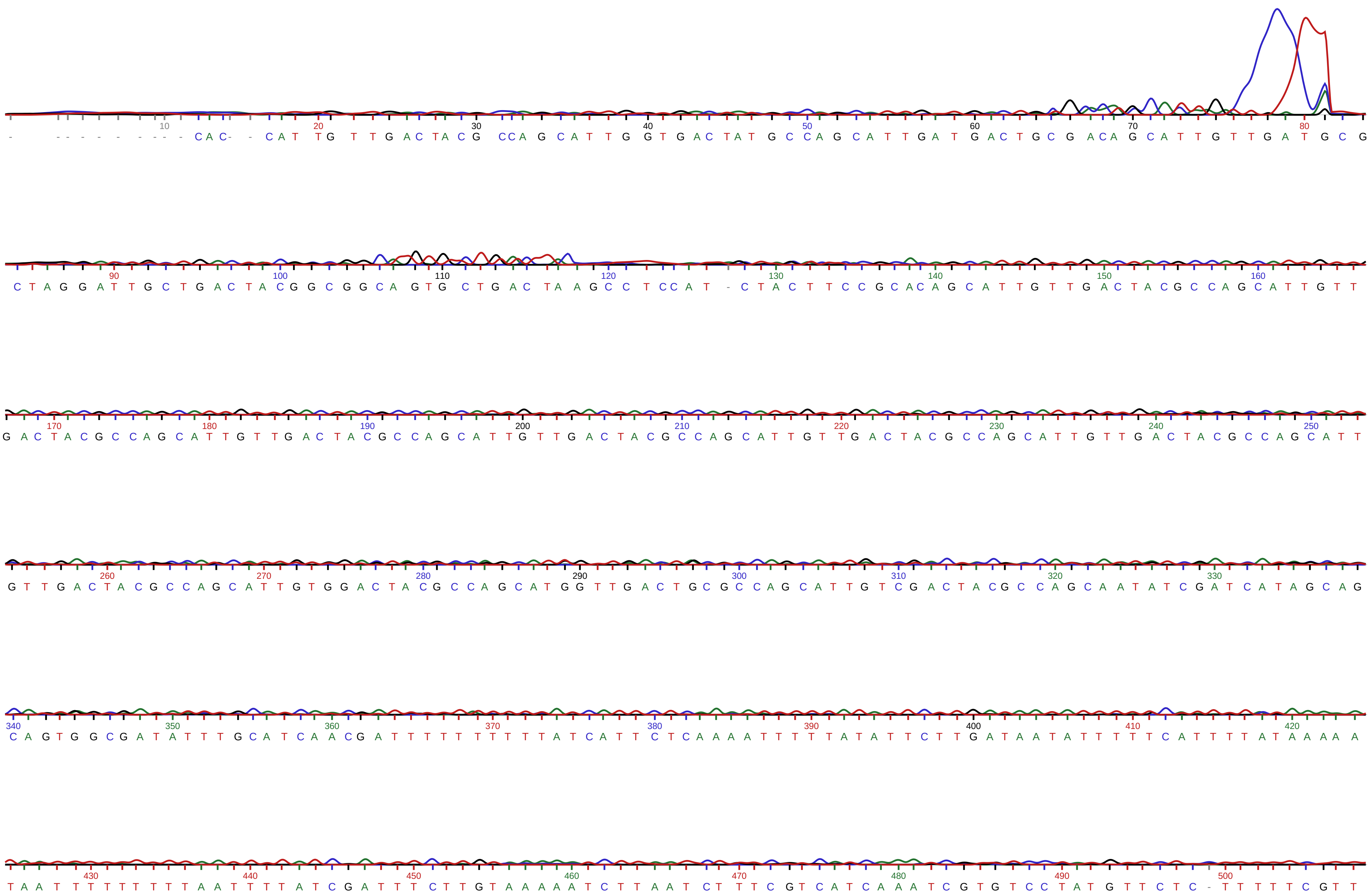

Samples: 11939
Bases: 930
Average spacing: 13

510 520 530 540 550 560 570 580 590

T G T T A T T T G - T T A T A A C C A T T A G A G G T G G G A G T A T C T A A T T T C T T T C T T T C A T C A T G T A A T T T C T G A T C T T C A C T A T T T T C A C G

600 610 620 630 640 650 660 670

G T T T T C G A A T A C T C T G G A C A A T C - - A C T C T T - C C A T T T C T A G T T C T T - - - T A T C T G T A A A T A A T C T A T C T T T T T C T C G A A T G A G

680 690 700 710 720 730 740 750

A T T C A T T T G T G T C T T T T T G A C C G A T T A A T A A A T G C A C T C C T A A A T C A C C T A C C A T A T T G T C A A A A T C A C T A G C A T C A G T A A A T

760 770 780 790 800 810 820 830 840

C C G G T T C C A T A A T T T G C T T T C G T A G A G A - C - - T T - A T T A T T T A C G G G T G G A A A A T T T - - - A A A T T G G - A A T T - - C - A T - - T C C A

850 860 870 880 890 900 910 920

T T A - A A G - T T T G G C T G C G T C T G A A A A A A T C C T - - - - T T T T G G G T G - - - - - C C G A A A T T G G G T G G A G A A A T T G A G A - - A - T G - G T G A G
